# Pandemic Fatigue and Anxiety Sensitivity as Associated Factors With Posttraumatic Stress Symptoms Among University Students in South Korea During the Prolonged COVID-19 Pandemic

**DOI:** 10.3389/ijph.2022.1604552

**Published:** 2022-05-13

**Authors:** Hee Jun Kim, Timothy J. Meeker, Ingrid K. Tulloch, Jake Mullins, Jin-Hee Park, Sun Hyoung Bae

**Affiliations:** ^1^ College of Nursing, Research Institute of Nursing Science, Ajou University, Suwon, South Korea; ^2^ Department of Neurosurgery, Johns Hopkins Medical Institute, Baltimore, MD, United States; ^3^ Department of Psychology, Morgan State University, Baltimore, MD, United States

**Keywords:** COVID-19, university students, pandemic fatigue, anxiety sensitivity, posttraumatic stress symptoms

## Abstract

**Objectives:** The global impact of COVID-19 driven by new variants may add to the negative mental health consequences of the prolonged pandemic, including posttraumatic stress symptoms (PTSS). University students may be prone to develop a series of PTSS due to life plan disruptions as well as increased uncertainty caused by the pandemic. The purpose of this study was to assess the associations between pandemic fatigue, anxiety sensitivity (AS), and PTSS among university students in South Korea.

**Methods:** Using convenience sampling, 400 students participated in this cross-sectional online survey. Descriptive statistics and linear mixed models were used to examine factors associated with PTSS.

**Results:** About one-third (32.3%) of the participants reported clinically significant levels of PTSS. Multivariate analyses revealed that pandemic fatigue (*β* = 0.124, *p* < 0.001) and AS (*β* = 0.212, *p* < 0.001) were significantly associated with PTSS while controlling for other study variables.

**Conclusion:** Young adults who feel more fatigue related to the COVID-19 pandemic and with high AS should be given access to mental health resources to better manage their mental health and reduce PTSS.

## Introduction

The novel coronavirus outbreak began in December of 2019, and the Corona virus diseases (COVID-19) pandemic persists worldwide. In January of 2020, the World Health Organization (WHO) declared a public health emergency [1]. Since this time, the number of new cases and deaths from COVID-19 has steadily increased, with 212 billion new cases and 4.4 million deaths as of August 2021 [2]. The global fourth wave of the COVID-19 appears driven by the Delta SARS-CoV-2 variant which was identified as more infectious, and with increased transmissibility even in vaccinated individuals [2].

Extensive consequences of the pandemic have been reported, influencing healthcare [3], education [4], the economy [5], and individuals’ daily lives [6]. Negative consequences of the pandemic on mental health outcomes have been widely studied, including associations with depression, anxiety disorders, stress, panic attacks, and posttraumatic stress symptoms (PTSS) [7, 8]. Young adults, in particular, are at high-risk for the development of mood and anxiety disorders [9] and the disruptions caused by the COVID-19 pandemic on educational and occupational opportunities may have increased their mental health vulnerability [10]. The various pandemic-mitigating strategies of social distancing, university closures, and remote classes have created mental health challenges for university students worldwide [11–17]. Furthermore, the fourth wave of SARS-CoV-2 strains has enhanced the threat toward individuals by extending uncertainty about when the pandemic will end. Having experienced and witnessed the unpredictable and uncontrollable attack of COVID-19 on life and the public policy efforts to control and manage public health emergencies, young adults may prone to develop a series of trauma-relevant psychological symptoms, namely PTSS [18].

Pandemic fatigue is a mental and physical phenomenon that occurs during a pandemic as a consequence of disruptions in individuals’ daily lives, such as lockdown, quarantine, and social distancing [19]. Pandemic fatigue is understood as a natural and expected response to a prolonged public health emergency, recent studies have reported evidence of pandemic fatigue after a few months of the lockdown implementation [20, 21]. Recently, Labrague and Ballad [22] found that a significant proportion of university students experience pandemic fatigue. Fatigue has also been associated with stress, general worries, and COVID-19 specific worries [21]. Inadequate physical/mental self-care among individuals increases fatigue, specifically during the prolonged pandemic, and the negative influence of pandemic fatigue on mental health has been reported [22].

Several personality traits are associated with various aspects of mental health. One such factor is anxiety sensitivity (AS), which is defined as the fear that anxiety symptoms or arousal, can have harmful consequences [23, 24]. The influence of AS on PTSS is of great concern regarding university students [25, 26]. Previous studies revealed that AS is a significant factor associated with PTSS [27, 28], and there are positive relationships between AS and posttraumatic stress disorder (PTSD) severity in military veterans [29], victims of domestic violence [30], and the general population [31]. Elevated trait AS was positively correlated with worry and fear of epidemic outbreaks during the Zika virus and Ebola virus epidemics [32, 33]. Individuals with a tendency to have greater “fear of fear” may experience greater negative feelings during the prolonged COVID-19 pandemic, negatively impacting their mental health.

In South Korea, the fourth wave of COVID-19 began in July of 2021. The number of daily new cases of COVID-19 approached its highest peak since the pandemic began (from 1.02 new cases per million in July 2020 to 29.76 in July 2021), possibly fueled by the increased rate of Delta variant transmission [34, 35]. Activities in universities are restricted during the fourth wave of pandemic in South Korea, and most of the classes are still conducted remotely. COVID-19 full vaccination rate was 12.3% in Mid-July 2021 because vaccines were not sufficiently available by that time in South Korea [36], compared to 48.9% in the United States [37]. Young adults who sought vaccination, especially during the prolonged pandemic, may be frustrated by the unavailability of the vaccines. Low vaccination rates combined with spread of the Delta Variant in South Korea this may exacerbate anxiety and stress associated with the COVID-19 pandemic [38].

## Methods

### Aim, Design, and Participants

To our knowledge, no current study has focused explicitly on pandemic fatigue, AS, and PTSS related to COVID-19 among university students. The purpose of this study was to describe the status of COVID-19 related pandemic fatigue and PTSS among university students in South Korea. We also examined the associations of pandemic fatigue and AS, along with COVID-19 related factors with PTSS in this population. A descriptive-correlational research design was used. Participants in this study were adults 18 years of age or older who lived in South Korea during the COVID-19 pandemic, were currently attending three or 4-year colleges and were native speakers of the Korean language. The number of study subjects was calculated using the G-Power program, with an effect size of 0.1, a significance level of 0.05, a power of 0.95, and 26 predictors required for multiple regression analysis. As a result, at least 358 subjects were required. Considering the dropout rate of 10%, 406 questionnaires were collected. After excluding 6 insincere questionnaires, 400 were included in the analysis. Participant students were from universities located in a diverse area in South Korea, including Seoul (the capital) and 9 provinces, with most of the participants (95.5%) from the large metropolitan areas of Seoul and K province.

### Procedure

We distributed the online survey using Google^®^ Forms between June 2021 and July 2021 to assess pandemic fatigue, AS, and PTSS during the COVID-19 pandemic. The survey link was published on university websites and online community dashboards. The Everytime (https://everytime.kr) is an online anonymous community dashboard for college students belonging to 400 universities in South Korea. It provides general information about college life, such as school life and academic management. Advertised universities include Ajou University, Gachon University, Yonsei University, Seoul National University, Suwon Science College, Dongguk University, Sungkyunkwan University, Hongik University, Korea National University of Arts, Soongsil University, Dongnam Health University, The University of Suwon, Duksung Women’s University, Dankook University, Seoul National University of Science and Technology, and Kookmin University. Information about the study was also posted on institutional homepages or on-campus bulletin boards (e.g., student halls, cafeterias, cafes, libraries, or on the student union hall) for recruitment. The study announcement described the purpose, method, and selection criteria of the study. A detailed explanation of the research was provided in a separate explanatory note, including a clause that there would be no disadvantages due to participation or non-participation in the research. We provided a QR code to access detailed information regarding the study and survey, and potential participants accessed the survey link through the QR code once they agreed to participate. Because of the nature of the online survey, voluntary participation was considered as consent to enroll in the study. Approximately 20–25 min were required to compete the survey; the survey was designed to proceed to the next item when all the previous items were completed to enhance the quality of the responses. Study participants were provided with mobile gift cards ($5) after the completion. The questionnaire consisted of two sections which investigated 1) demographics and COVID-19 related characteristics, 2) pandemic fatigue, AS, and PTSS.

### Measures

#### Posttraumatic Stress Symptoms

We used the Impact of Event Scale-6 (IES-6), which is a 6-item instrument to assess PTSS. This is an abbreviated version of the Impact of Event Scale-revised (IES-R) whose validity has been previously reported [39, 40]. It includes three symptom subscales of posttraumatic stress disorder (PTSD), including intrusion, hyper-arousal, and avoidance. Respondents were asked to report how distressed or bothered they were, over the past 7 days, by trauma-related symptoms ascribed to the COVID-19 pandemic. Each item is scored 0 (not at all) to 4 (extremely). The score was calculated as the average of 6 items, with a higher total score indicating a higher level of PTSS. For this study, the English version of the IES-6 was translated into Korean by two Korean-English bilingual translators. These translators (HJK and JYH) were native Koreans, received a doctoral degree in the US and had practiced in academia in the US. Any differences were reconciled, and the reconciled version was translated back into English by an independent, bilingual translator. The back-translated English version was compared with the original English version to check for any loss of meaning. Cronbach’s alpha [41] for the IES-6 in the current study was 0.82.

#### Pandemic Fatigue

Pandemic fatigue was measured using the Pandemic Fatigue Scale (PFS) developed by Lilleholt et al. [42]. Translation of the PFS into the Korean language was conducted in the same manner as described above to translate the IES-6. The PFS consists of six items that assess pandemic fatigue, defined as demotivation to engage in recommended COVID-19 protective behaviors (behavioral fatigue) and resistance to receiving information meant to mitigate the spread of the pandemic (information fatigue). Behavioral fatigue items included statements such as “When friends or family members talk about COVID-19, I try to change the subject because I do not want to talk about it anymore.” Information fatigue items include statements such as “I am sick of hearing about COVID-19.” For each statement, participants choose a response score ranging from 1 to 7 to indicate that they “strongly disagree” to “strongly agree” with the statement. The sum of the response scores for all six items provides a measure of participants’ overall pandemic fatigue. The higher the overall PFS score, the greater the pandemic fatigue reported by participants. Cronbach’s alpha for the PFS in the current study was 0.84.

#### Anxiety Sensitivity

The Anxiety Sensitivity Index (ASI) is a tool to measure the tendency of excessive and persistent responses to anxiety-triggering stimuli [43]. In this study, the Korean version of ASI was used to measure AS. The ASI is an 18-item self-report tool for assessing anxiety-related physical, cognitive, or social concerns. Respondents indicated their level of agreement with each item on a scale ranging from “very little” (coded as 0) to “very much” (coded as 4). The total score is the sum score of all items, with higher scores on the scale representing more severe AS. The Korean version of ASI has been verified for validity and reliability [44]. In this study, Cronbach’s alpha for the ASI was 0.85.

#### Demographics and COVID-19 Related Characteristics

Demographics collected consisted of questions about age, gender, education, perceived economic status, prescribed or over-the-counter (OTC) medications, frequency of physical activity, tobacco smoking, alcohol consumption, and caffeine intake. COVID-19-related characteristics consisted of questions about COVID-19-related health behaviors (wearing protective equipment, hand washing, social distancing, etc.). Participants were also asked about COVID-19 related symptoms and COVID-19 PCR diagnostic testing, COVID-19 vaccination status, their intention to be vaccinated, and levels of worry related to the vaccine (measured using a 5-point Likert scale where 1 indicates not at all worried and 5 indicates extremely worried) and COVID-19 exposure.

### Analysis

Participant demographics and COVID-19-related characteristics, pandemic fatigue, AS, and PTSS levels were analyzed as descriptive statistics (*n*, %, mean, SD, range). Normality of the data was checked using histograms and normal probability plots, skewness, or kurtosis measures. An independent t-test or a one-way ANOVA was performed for differences in PTSS according to the participants’ demographics and COVID-19-related stressors. Pearson’s correlation coefficients were used to analyze the correlation among study variables, including pandemic fatigue, AS, and PTSS. The Benjamini-Hochberg procedure was used to control the false discovery rate for multiple comparisons in the bivariate analysis.

Linear mixed models nested by location of university were used to examine factors associated with PTSS. All variables designated as associated with PTSS in bivariate analysis were entered into the models along with several demographic characteristics (gender, major, education, and perceived economic status). Given the strong evidence of gender differences in the mental health characteristics during the COVID-19 pandemic [45, 46], we conducted a subgroup analysis by gender. Six out of 406 participants were excluded due to incomplete responses, and there were no missing data in the dataset. Multicollinearity was evaluated using VIF, residuals, and outliers to determine whether the independent variables were suitable for regression analysis. Data were analyzed using the STATA 15.1 (StataCorp, Texas, United States).

## Results

### Sample Characteristics Related to COVID-19

The average participant age was 21.7 years (SD = 2.6, range: 18–39 years). A majority were female (71.8%, *n* = 287) and attending a 4-year college (82.8%, *n* = 331). Fifty-two percent of the participants were attending a university located in Seoul, and 42.5% in K province the location of two metropolitan areas in South Korea. Academic fields of study of participating students were diverse, including basic science and engineering (39.5%), social science, humanities, and education (42.3%), and medical (11.5%). Almost half of the participants reported they were “satisfied” with their economic status (46.5%, *n* = 186). More than 70% of participants did not take any prescribed (86.0%, *n* = 344) or OTC medications (74.3%, *n* = 297), while 12.5% were currently smoking. The percent of participants reporting not consuming alcohol or caffeine was 23.8% and 13.8%, respectively (Table 1).

**TABLE 1:** General characteristics of participants (N = 400) (Mental health status during the COVID-19 pandemic among university students, South Korea, 2021).

Characteristics	n (%)
Gender	
Male	113 (28.2)
Female	287 (71.8)
Age, Mean (SD), range	21.7 (2.6), 18–39
Location of university	
Seoul	211 (52.8)
K province	170 (42.5)
Else	19 (4.7)
Major	
Basic science and Engineering	158 (39.5)
Social science, Humanities and Education	169 (42.3)
Arts & Physical	27 (6.7)
Medical	46 (11.5)
Education	
Three-year college	53 (13.2)
Four-year college	331 (82.8)
Else	16 (4.0)
Perceived economic status	
Satisfied	186 (46.5)
Neither Satisfied nor Dissatisfied	88 (22.0)
Dissatisfied	126 (31.5)
Frequency of physical activity (last week)	
0	44 (11.0)
1–2 times	152 (38.0)
3–5 times	132 (33.0)
6–7 times	50 (12.5)
More than 7 times	22 (5.5)
Prescribed medication	
Yes	56 (14.0)
No	344 (86.0)
OTC medication	
Yes	103 (25.7)
No	297 (74.3)
Smoking	
Never smoked	309 (77.3)
Stopped smoking	41 (10.2)
Currently Smoking	50 (12.5)
Drinking	
No drinking	94 (23.8)
1–2 drinks per month	168 (42.5)
More than 1–2 drinks per week	133 (33.7)
Caffeine intake	
No caffeine	55 (13.8)
Less than 6 drinks per week	235 (58.9)
6 drinks or more per week	109 (27.3)

Abbreviation: OTC, over the counter; SD, standard deviation.

Fifty-six percent of participating university students reported staying at home most of the time or always to avoid infection. Most participants (95.9%) always wore a mask when going outside, and KF-94 masks specifically produced to avoid droplet infection were the most commonly used mask (58.2%). Fifty-six percent of participants watched news about COVID-19 for 1, 2 h per week. More than half (55.0%) of the participants had COVID-19-like symptoms in the past week, including coughing, chills, or runny nose. Approximately twenty-five percent of the participants had COVID-19 test, and all were negative. Most participants worried about COVID-19 exposure (75.3%) and going outside (68.8%) at moderate or higher levels. At the time of the survey, only 13% of the participants were vaccinated, but 85.3% of non-vaccinated participants answered they are likely or definitely getting vaccinated when it’s available. About fifty percent of participants were at least moderately worried about side effects of COVID-19 vaccines (Table 2).

**TABLE 2:** COVID-19 related characteristics of participants (N = 400) (Mental health status during the COVID-19 pandemic among university students, South Korea, 2021).

Characteristics	n (%)
Stay home to avoid infection	
Never–Some of the time	103 (25.8)
About half of the time	73 (18.3)
Most of the time–Always	224 (56.0)
Wearing a mask when going outside Never	
Never	1 (0.3)
Some of the time	1 (0.3)
About half of the time	1 (0.3)
Most of the time	13 (3.2)
Always	384 (95.9)
Mask type	
KF94 mask	233 (58.2)
N95 respirator	5 (1.2)
Medical mask	147 (36.7)
Cloth mask	13 (3.2)
Scarf or bandana	1 (0.3)
None	1 (0.3)
Frequency of washing hands	
Less than twice a day	63 (15.7)
3–6 times a day	195 (48.8)
More than 7 times a day	142 (35.5)
Frequency of sanitizing residence	
Not at all	48 (12.0)
Once per week	196 (49.0)
More than once per day	156 (39.0)
Frequency of watching news about COVID-19	
Not at all	76 (19.0)
1–2 h per week	227 (56.8)
More than 3–5 h per week	97 (24.2)
Working at home	
None–A little	78 (19.5)
About half	112 (28.0)
Most of it–All of it	210 (52.5)
COVID-19 like symptoms	
Yes	220 (55.0)
No	180 (45.0)
Test COVID-19	
Yes	106 (26.5)
No	294 (73.5)
Worried about COVID-19 exposure	
Not at all–A little bit	99 (24.7)
Moderately	131 (32.8)
Quite a bit–Extremely	160 (42.5)
Worried about going outside e bit	
Not at all–A littl	125 (31.2)
Moderately	136 (34.0)
Quite a bit–Extremely	139 (34.8)
Get COVID-19 vaccinated	
Yes	52 (13.0)
No	348 (87.0)
Worried about side effects of vaccines	
Not at all–A little bit	194 (48.5)
Moderately	113 (28.3)
Quite a bit - Extremely	93 (23.2)
Willingness of getting vaccinated^a^	
Unlikely	51 (14.7)
Likely	140 (40.5)
Definitely	155 (44.8)

Abbreviation: COVID-19, Coronavirus disease.

a Responses from non-vaccinated participants, excluding non-response (*n* = 346).

### Levels of PTSS, Pandemic Fatigue, and Anxiety Sensitivity

The average of PTSS of the participants was 14.06 (SD = 5.04) and ranged from 6 to 29. Using the cutoff score of 10 previously validated for the IES-6 scale [39, 40], 32.3% of the participants self-reported levels of PTSS indicative of PTSD. The average total pandemic fatigue score was 22.27 (SD = 7.41), ranging from 6 to 40. The anxiety sensitivity level of the participants averaged 11.27 (SD = 8.75), ranging from 0 to 52 (Table 3).

**TABLE 3:** Correlation among study variables (N = 400) (Mental health status during the COVID-19 pandemic among university students, South Korea, 2021).

Variables	r (*p*)		
	Posttraumatic stress symptoms	COVID-19 fatigue	Anxiety sensitivity
Posttraumatic stress symptoms	—		
COVID-19 fatigue	0.171 (0.001)	—	
Anxiety sensitivity	0.413 (<0.001)	0.055 (0.270)	—
Mean	14.06	22.27	11.27
Standard deviation	5.04	7.41	8.75
Range	6–29	6–40	0–52

Abbreviation: COVID-19, Coronavirus disease.

### Factors Associated With PTSS

Demographic and COVID-19 related factors associated with PTSS in the bivariate analysis included education, having been taking OTC/prescribed medication, staying home to avoid infection, mask type, having had COVID-19 like symptoms, worry about COVID-19 exposure, worry about going outside, and worry about side effects of COVID-19 vaccines (Supplementary S1–S3). Among these variables, mask type was excluded in the final model due to its low level of variance.

The linear mixed model (Table 4), nested by location of university, revealed that staying at home to avoid infection (*β* = 2.345, *p* < 0.001, 95% CI = [1.034, 3.656]), being worried about COVID-19 exposure (*β* = 1.404, *p* = 0.050, 95% CI = [0.003, 2.805]), COVID-19 fatigue (*β* = 0.124, *p* < 0.001, 95% CI = [0.062, 0.186]), and AS (*β* = 0.212, *p* < 0.001, 95% CI = [0.158, 0.265]) were significantly associated with PTSS after controlling for other demographic and COVID-19 related factors. Those who stayed at home about half of the time to avoid infection were estimated to be 2.3 units higher in PTSS compared to those who never or sometimes stayed home. Those who worried quite a bit or extremely about COVID-19 exposure were estimated to be 1.4 units higher in PTSS compared to those who worried not at all or a little bit. Higher COVID-19 fatigue and AS was associated with higher PTSS. Subgroup analysis by gender indicated that COVID-19 fatigue (*β* = 0.237, *p* < 0.001, 95% CI = [0.113, 0.360]) and AS (*β* = 0.262, *p* < 0.001, 95% CI = [0.139, 0.375]) were significantly associated with PTSS in males, while staying at home to avoid infection (*β* = 2.874, *p* = 0.001, 95% CI = [1.208, 4.540]), being worried about going outside due to COVID-19 (staying at home to avoid infection (*β* = 1.988, *p* = 0.029, 95% CI = [0.207, 3.768]), COVID-19 fatigue (*β* = 0.086, *p* = 0.017, 95% CI = [0.015, 0.157]), and AS (*β* = 0.208, *p* < 0.001, 95% CI = [0.148, 0.267]) were significantly associated with PTSS in females (Table 4).

**TABLE 4:** Linear mixed models to test associated factors to Posttraumatic Stress Symptom (N = 400) (Mental health status during the COVID-19 pandemic among university students, South Korea, 2021).

	Total				Subgroup analysis							
					Males				Females			
	*β*	SE	*p*	95% CI	*β*	SE	*p*	95% CI	*β*	SE	*p*	95% CI
Gender (female)	‒0.458	0.541	0.397	‒1.518, 0.602								
Major												
Science and Engineering (ref)												
Education, Social Science, and Humanities	0.265	0.491	0.590	‒0.698, 1.227	0.406	0.871	0.641	‒1.301, 2.114	0.214	0.575	0.709	‒0.912, 1.340
Arts and Physical	1.286	0.906	0.156	‒0.489, 3.061	3.538	1.919	0.065	‒0.224, 7.299	1.096	1.017	0.281	‒0.897, 3.089
Medical	0.117	0.781	0.881	‒1.413, 1.647	3.005	2.285	0.189	‒1.474, 7.485	‒0.119	0.818	0.884	‒1.721, 1.483
Education												
2 or 3 years college (ref)												
4 years college	‒0.860	0.606	0.156	‒2.047, 0.327	‒1.009	1.203	0.401	‒3.366, 1.348	‒0.718	0.699	0.305	‒2.088, 0.653
Perceived economic status												
Satisfied (ref)												
Neutral or dissatisfied	‒0.112	0.138	0.418	‒0.382, 0.159	‒1.646	0.843	0.051	‒3.298, 0.006	‒0.387	0.526	0.462	‒1.417, 0.643
Prescribed medication (Yes)	‒0.187	0.637	0.769	‒1.435, 1.061	2.283	1.208	0.059	‒0.084, 4.651	‒0.121	0.739	0.129	‒2.570, 0.327
OTC medication (Yes)	0.486	0.502	0.334	‒0.499, 1.471	0.528	1.043	0.613	‒1.516, 2.571	0.362	0.567	0.523	‒0.749, 1.473
Stayed at home to avoid infection												
Never–Some of the time (ref)												
About half of the time	2.345	0.669	<0.001	1.034, 3.656	2.152	1.125	0.056	‒0.054, 4.357	2.874	0.850	0.001	1.208, 4.540
Most of the time–Always	0.871	0.544	0.109	‒0.195, 1.938	1.422	0.987	0.150	‒0.513, 3.356	0.824	0.652	0.206	‒0.453, 2.101
COVID-19 like symptoms (Yes)	0.565	0.452	0.212	‒0.321, 1.450	1.230	0.842	0.144	‒0.421, 2.881	0.270	0.527	0.068	‒0.764, 1.304
Worried about COVID-19 exposure												
Not at all–a little bit (ref)												
Moderately	0.297	0.630	0.637	‒0.937, 1.531	‒0.470	0.988	0.634	‒2.407, 1.467	0.336	0.817	0.681	‒1.266, 1.938
Quite a bit–Extremely	1.404	0.715	0.050	0.003, 2.805	1.962	1.262	0.120	‒0.511, 4.435	1.288	0.892	0.149	‒0.461, 3.036
Worried about going outside due to COVID-19												
Not at all–a little bit (ref)												
Moderately	‒0.148	0.613	0.810	‒1.350, 1.055	‒1.518	0.988	0.124	‒3.453, 0.418	0.653	0.792	0.410	‒0.899, 2.205
Quite a bit–Extremely	1.099	0.739	0.137	‒0.350, 2.548	‒1.752	1.450	0.227	‒4.593, 1.090	1.988	0.908	0.029	0.207, 3.768
Worried about side effects of COVID-19 vaccines												
Not at all–a little bit (ref)												
Moderately	0.355	0.526	0.500	‒0.676, 1.387	0.060	1.024	0.953	‒1.948, 2.068	0.416	0.610	0.495	‒0.780, 1.612
Quite a bit–Extremely	0.833	0.569	0.144	‒0.283, 1.949	1.483	1.146	0.196	‒0.763, 3.729	0.860	0.650	0.186	‒0.414, 2.133
COVID-19 fatigue	0.124	0.032	<0.001	0.062, 0.186	0.237	0.063	<0.001	0.113, 0.360	0.086	0.036	0.017	0.015, 0.157
Anxiety Sensitivity	0.212	0.027	<0.001	0.158, 0.265	0.262	0.063	<0.001	0.139, 0.385	0.208	0.030	<0.001	0.148, 0.267

Abbreviation: CI, confidence intervlas; COVID-19, Coronavirus disease; SE, standard error.

## Discussion

This study examined COVID-19 related PTSS and its associated factors, including demographic characteristics, COVID-19 related factors, pandemic fatigue, and AS among university students in South Korea. The prevalence of PTSS indicative of probable PTSD in our sample was 32.3%, which is relatively high compared to another study in China conducted 1 month after the December 2019 COVID-19 outbreak [11]. A recent study among healthcare workers during the COVID-19 pandemic reported a PTSS prevalence of 26.2% in Italy [47]. Part of the reason for this discrepancy may be the use of different measurements for PTSS between the two studies; the previous studies used PTSD Checklist for DSM-5, whereas the current study used the IES-6 scale. Our results are comparable to a study conducted in the US, reporting 31.8% of prevalence of PTSS indicative of likely PTSD among young adults [11]. These results suggest a relatively high prevalence of PTSD-like symptomatology among young adults during the most recent wave of the COVID-19 pandemic. University students may be particularly distressed in managing their school or work responsibilities, while uncertainty about the end of the COVID-19 pandemic may exacerbate adverse effects on their mental health.

In the linear mixed model analysis, several COVID-19 related factors were associated with PTSS, including staying at home to avoid infection, and worry regarding exposure to SARS-CoV-2 virus. As most participants were living in an urban area where the COVID-19 infection rates were high in South Korea, they may have been worried about viral infection and experienced stress from more restrictive social distancing policies. Interestingly, subgroup analysis revealed some gender differences in those COVID-19 related factors that were associated with PTSS; for male students, the two factors (staying at home to avoid infection and worry regarding exposure to SARS-CoV-2 virus) were not significantly associated with PTSS, whereas staying at home to avoid infection and worry about going outside due to COVID-19 were significantly associated with PTSS in females. These differences suggest that female students are more sensitive to quarantine methods (e.g., staying at home, worrying about going outside) to avoid infection and that these quarantine measures are associated with higher PTSS in females.

The current study revealed that pandemic fatigue and AS were significant factors of PTSS among both male and female university students. Pandemic fatigue is found across cultures and populations [42, 48] as efforts to reduce the spread of COVID-19 are ongoing. The pandemic fatigue phenomena might be related to social and economic factors as well as other pre-existing risks [42, 48, 49]. Morgul et al. [48] for example, reported a significant proportion of participants with less knowledge about COVID-19, general educational attainment, and lower socioeconomic status had greater pandemic fatigue than individuals not belonging to those groups. Furthermore, Lilleholt et al. [42] found greater pandemic fatigue in German and Danish individuals who reported greater worry about the economic consequences of the pandemic. The current study adds to the literature by providing evidence on pandemic fatigue among young adults with relatively self-rated high socio-economic status.

Young adults who feel more fatigue from the COVID-19 pandemic may be more vulnerable to PTSD-like symptoms as their physical and psychological resources are taxed during mandated restrictions on activities to mitigate against viral exposure and spread. The increased psychological burden caused by navigating additional public health safeguards associated with the COVID-19 pandemic is a likely contributing factor exacerbating PTSD-like symptomatology. Previously, pandemic fatigue was found to be an important concern for healthcare workers during the pandemic [50], and the current study results suggests pandemic-related fatigue issues should also be addressed in university students given the associations found between pandemic fatigue and mental health during the COVID-19 pandemic among this population.

AS is a factor for developing PTSD. Previous studies have reported enhanced AS as predictive of development of PTSD symptomatology [28, 51] and positive correlations have been reported between the IES-R and AS [52]. More recent research has shown AS to be related to COVID-19-related anxiety, COVID-19-related worry, overall anxiety severity, depression, psychological impairment in university populations, health care workers and the general population in several countries [52–57].

The current study indicated AS was the factor explaining the amount of highest variance in PTSS among university students during the COVID-19 pandemic. This is consistent with a previous study reporting AS is predictive of COVID-19-related distress and worry among Canadian and American adults [58, 59]. The current study supports the influence of AS on COVID-19 PTSD-like symptomatology among young adults. The results of current study indicate that personality traits, such as AS, influence the PTSS more than demographic characteristics or even COVID-19 specific worries among Korean university students. University students with high AS should be screened and be provided with additional resources to better manage their mental health and to reduce the risk of developing PTSD.

In line with previous findings [11–17], the current study found negative effects of the COVID-19 pandemic on university students’ mental health status. To reduce the damaging mental health influences observed during the pandemic in this population, both local (university) and national level public policies should be developed. At local level, universities may develop and incorporate various online-based wellness programs using telemedicine, boot camps for coping strategies, self-help mindfulness therapy, virtual group exercise and meditation/mindfulness sessions [12, 15]. At the national level, policies to support universities and students are necessary for providing timely and accurate epidemic information and adequate and uniform schooling regulations (e.g., online vs. face-to-face class options, remote management/counseling programs) to avoid any confusion and uncertainty aroused from inconsistent regulations during the pandemic period.

### Limitation

One limitation of this study is that we collected data only from South Korean university students in urban areas where relatively high COVID-19 transmission exists. Also, risk of selection bias and response-set bias may have occurred due to the convenience sampling method and data collection using an online/self-reported format. Therefore, generalizability of our results is limited. The scales used to measure AS and pandemic fatigue have not previously been used or validated among South Koreans. However, the undertook a thorough translation process and the internal consistency of the scales from the current sample was adequate. Future studies validating these measures among South Korean population are needed. Other important factors affecting mental health among university students, including self-rated health status, and life-style such as spending times outside or on electronic screens [15] were not captured in this study. Lastly, the incidence, pattern, and mutation of SARS-CoV-2 is dynamic; therefore, it is necessary to longitudinally examine changes in PTSD-like symptomatology related to the COVID-19 pandemic among university students.

### Conclusion

Covid-19 pandemic has been prolonged with new variants (e.g., Delta, Omicron), and their impact on mental health should be recognized. Approximately one-third of the university students reported having experienced PTSS related to COVID-19 indicative of clinical PTSD. Pandemic fatigue and AS was significantly associated with PTSS in this population. Based on the results of the current study, we suggest university-wide programs to enhance mental health status among students, including providing platforms of social networking and interactions with peers to share their thoughts and emotions. Using various forms of digital and other interactions, including messaging or calls using social media has been reported to be associated with better mental health among university students [60]. Easily accessible online counseling for students who need the service should be provided, especially during the pandemic. AS was previously reported associated with suicidal ideation and past suicide attempts [24], and more attention should be given regarding this issue among Korean university students during the COVID-19 pandemic. Based on the previous evidence on efficacy of brief cognitive interventions on AS reduction [61], we also suggest providing interventions through convenient therapy platforms, such as video-based intervention [62], online interactions using social media and email/phone calls to foster bonding and social connection [15] to reduce AS among vulnerable university students.

## Supplementary Material

Supplementary Table S1 Difference of posttraumatic stress symptoms according to general characteristics of participants (N = 400) (Mental health status during the COVID-19 pandemic among university students, South Korea, 2021).

Supplementary Table S2 Difference of posttraumatic stress symptoms according to COVID-19 related characteristics of participants (N = 400) (Mental health status during the COVID-19 pandemic among university students, South Korea, 2021).

Supplementary Table S3 Benjamini-Hochberg procedure results of the bivariate analysis results (N = 400) (Mental health status during the COVID-19 pandemic among university students, South Korea, 2021).

Supplementary Table S4 Normal probability, histogram of the PTSS (dependent variable), and skewness and kurtosis, VIF of the study variables (Mental health status during the COVID-19 pandemic among university students, South Korea, 2021).

## Abbreviations

AS, anxiety sensitivity; IES-6, Impact of Events Scale; PTSS, posttraumatic stress symptoms.
