# Supplementary material for: Pandemic Fatigue and Anxiety Sensitivity as Associated Factors With Posttraumatic Stress Symptoms Among University Students in South Korea During the Prolonged COVID-19 Pandemic
Source: Int J Public Health. 2022 May 13;67:1604552. doi: 10.3389/ijph.2022.1604552 (PMC9137407; doi:10.3389/ijph.2022.1604552)
Supplement: Supplementary file 1 [file DataSheet1.docx]

**Supplementary 1. Difference of posttraumatic stress symptoms according to general characteristics of participants (South Korea, 2021)**

| **Characteristics** | **N** | **Mean±SD** | **t or F** | ***p*** |
| --- | --- | --- | --- | --- |
| Gender |  |  |  |  |
| Male  Female | 113  287 | 13.91±5.26  14.12±4.96 | -0.38 | .708 |
| Education |  |  |  |  |
| Three-year college  Four-year college | 69  331 | 15.33±5.13  13.80±5.00 | 2.31 | .021 |
| Perceived economic status |  |  |  |  |
| Satisfied  Neither Satisfied nor Dissatisfied  Dissatisfied | 186  88  126 | 13.75±4.81  14.13±5.02  14.48±5.40 | 7.81 | .459 |
| Regular physical activity |  |  |  |  |
| Less than 2 times  3-5 times  More than 6 times | 196  132  72 | 13.90±5.13  14.57±5.32  13.57±4.22 | 1.11 | .332 |
| Taking prescribed medication |  |  |  |  |
| Yes  No | 56  344 | 15.23±5.88  13.87±4.88 | 1.88 | .061 |
| Taking over-the-counter medication |  |  |  |  |
| Yes  No | 1033  297 | 14.96±4.95  13.75±505 | 2.11 | .036 |
| Smoking |  |  |  |  |
| Never smoked  Stopped smoking  Currently smoking | 309  41  50 | 13.85±5.04  15.07±4.84  14.56±5.17 | 1.35 | .261 |
| Drinking |  |  |  |  |
| No drinking  1-2 drinks per month  More than 1-2 drinks per week | 94  168  133 | 14.70±4.51  13.72±5.38  14.08±4.98 | 1.14 | .321 |
| Caffeine intake |  |  |  |  |
| No caffeine  Less than 6 drinks per week  6 drinks or more per week | 55  235  109 | 14.62±5.18  13.69±4.95  14.64±5.14 | 1.72 | .181 |

**Supplementary 2. Difference of Posttraumatic Stress Symptoms according to COVID-19 related characteristics of participants (South Korea, 2021)**

| **Characteristics** | **n** | **Mean±SD** | **t or F** | ***p*** | **Post-hoc** |
| --- | --- | --- | --- | --- | --- |
| Stay home to avoid infection |  |  |  |  |  |
| Never or some of the time  About half of the time  Most of the time or always | 103  73  224 | 13.10±4.73  15.23±5.50  14.13±4.96 | 3.93 | .020 |  |
| Wearing a mask when going outside |  |  |  |  |  |
| Never  Some of the time  About half of the time  Most of the time  Always | 1  1  1  13  384 | 26.00  15.00  17.00  15.00±5.63  13.99±5.01 | 1.64 | .164 |  |
| Mask type |  |  |  |  |  |
| KF94 mask  N95 respirator  Medical mask  Cloth mask  Scarf or bandana  None | 233  5  147  1  1  1 | 14.27±4.97  14.60±3.51  13.45±4.88  15.00±6.23  28.00  26.00 | 3.36 | .005 |  |
| Frequency of washing hands |  |  |  |  |  |
| Less than twice a day  3-6 times a day  More than 7 times a day | 63  195  142 | 14.51±5.59  13.70±5.10  14.37±4.70 | 1.01 | .364 |  |
| Frequency of sanitizing residence |  |  |  |  |  |
| Not at all  Once per week  More than once per day | 48  196  156 | 15.33±5.61  13.85±5.05  13.94±4.82 | 1.75 | .174 |  |
| Frequency of watching news about COVID-19 |  |  |  |  |  |
| Not at all  1-2 hours per week  More than 3-5 hours per week | 76  227  97 | 14.14±5.98  13.80±4.85  14.61±4.67 | 0.88 | .415 |  |
| Working at home |  |  |  |  |  |
| None or a little  About half  Most of or all of it | 78  112  210 | 13.81±5.48  14.23±5.10  14.07±4.86 | 0.16 | .850 |  |
| COVID-19 like symptoms |  |  |  |  |  |
| Yes  No | 220  180 | 14.69±5.10  13.30±4.89 | 2.56 | .006 |  |
| Test COVID-19 |  |  |  |  |  |
| Yes  No | 106  294 | 14.41±5.19  13.94±4.99 | 0.82 | .415 |  |
| Worried about COVID-19 exposure |  |  |  |  |  |
| Not at all or a little bit ^a^  Moderately ^b^  Quite a bit or extremely ^c^ | 99  131  160 | 12.77±5.08  13.73±4.98  15.08±4.89 | 7.21 | .001 | a,b<c |
| Worried about going outside |  |  |  |  |  |
| Not at all or a little bit ^a^  Moderately ^b^  Quite a bit or extremely ^c^ | 125  136  139 | 13.27±5.33  13.19±4.71  15.63±4.75 | 10.75 | <.001 | a,b<c |
| Get COVID-19 vaccinated |  |  |  |  |  |
| Yes  No | 52  348 | 14.04±5.66  14.07±4.95 | -0.04 | .971 |  |
| Worried about side effects of vaccines |  |  |  |  |  |
| Not at all or a little bit ^a^  Moderately ^b^  Quite a bit or extremely ^c^ | 194  113  93 | 13.22±5.24  14.21±4.27  15.63±5.14 | 7.50 | .001 | a,b<c |

**Supplementary 3. Benjamini-Hochberg procedure results of the bivariate analysis results (South Korea, 2021)**

| Variable | P-Value | Rank | (i/m)*Q |
| --- | --- | --- | --- |
| Worried about going outside | <0.001 | 1 | 0.008 |
| COVID-19 fatigue | <0.001 | 1 | 0.008 |
| Worried about COVID-19 exposure | 0.001 | 3 | 0.025 |
| Worried about side effects of vaccines | 0.001 | 3 | 0.025 |
| Anxiety sensitivity | 0.001 | 3 | 0.025 |
| Mask type | 0.005 | 6 | 0.050 |
| COVID-19 like symptoms | 0.006 | 7 | 0.058 |
| Stay home to avoid infection | 0.020 | 8 | 0.066 |
| Education | 0.021 | 9 | 0.075 |
| Taking over-the-counter medication | 0.036 | 10 | 0.083 |
| Taking prescribed medication | 0.061 | 11 | 0.091 |
| Wearing a mask when going outside | 0.164 | 12 | 0.100 |
| Frequency of sanitizing residence | 0.174 | 13 | 0.108 |
| Caffeine intake | 0.181 | 14 | 0.112 |
| Smoking | 0.261 | 15 | 0.125 |
| Drinking | 0.321 | 16 | 0.133 |
| Regular physical activity | 0.332 | 17 | 0.142 |
| Frequency of washing hands | 0.364 | 18 | 0.150 |
| Frequency of watching news about  COVID-19 | 0.415 | 19 | 0.158 |
| Test COVID-19 | 0.415 | 19 | 0.158 |
| Perceived economic status | 0.459 | 21 | 0.175 |
| Gender | 0.708 | 22 | 0.183 |
| Working at home | 0.850 | 23 | 0.192 |
| Get COVID-19 vaccinated | 0.971 | 24 | 0.200 |

**Note.** i= rank of p-value, m = total number of tests, Q = 0.2 (accepting 20% false discovery rate)

**Supplementary 4. Normal probability, histogram of the PTSS (dependent variable), and skewness and kurtosis, VIF of the study variables. (South Korea, 2021)**

| Skewness and kurtosis, VIF of the study variables | | | |
| --- | --- | --- | --- |
|  | Skewness | Kurtosis | VIF |
| PTSS | .303 | 2.548 |  |
| COVID-19 fatigue | -.018 | 2.558 | 1.11 |
| Anxiety Sensitivity | 1.221 | 4.653 | 1.21 |
| Perceived economic status |  |  |  |
| Satisfied (ref) |  |  | - |
| Neutral or Dissatisfied |  |  | 1.24 |
| Prescribed medication (Yes) |  |  | 1.08 |
| OTC medication (Yes) |  |  | 1.05 |
| Stayed at home to avoid infection |  |  |  |
| Never - about half of the time (ref) |  |  | - |
| Almost - Always |  |  | 1.13 |
| COVID-19 like symptoms (Yes) |  |  | 1.10 |
| Worried about COVID-19 virus exposure |  |  |  |
| Not at all - a little bit (ref) |  |  | - |
| Moderately |  |  | 1.91 |
| Quite a bit - Extremely |  |  | 2.74 |
| Worried about going outside due to COVID-19 |  |  |  |
| Not at all - a little bit (ref) |  |  | - |
| Moderately |  |  | 1.75 |
| Quite a bit - Extremely |  |  | 2.60 |
| Worried about side effects of COVID-19 vaccines |  |  |  |
| Not at all - a little bit (ref) |  |  | - |
| Mod - Extremely |  |  | 1.15 |
